# Fine-Tuning Florigen Increases Field Yield Through Improving Photosynthesis in Soybean

**DOI:** 10.3389/fpls.2021.710754

**Published:** 2021-08-16

**Authors:** Kun Xu, Xiao-Mei Zhang, Haifeng Chen, Chanjuan Zhang, Jinlong Zhu, Zhiyuan Cheng, Penghui Huang, Xinan Zhou, Yuchen Miao, Xianzhong Feng, Yong-Fu Fu

**Affiliations:** ^1^MOA Key Laboratory of Soybean Biology, National Key Facility of Crop Gene Resource and Genetic Improvement, Institute of Crop Sciences, Chinese Academy of Agricultural Sciences, Beijing, China; ^2^Key Laboratory of Soybean Molecular Design Breeding, Northeast Institute of Geography and Agroecology, The Innovative Academy of Seed Design, Chinese Academy of Sciences, Harbin, China; ^3^Oil Crops Research Institute, Chinese Academy of Agricultural Sciences, Key Laboratory of Biology and Genetic Improvement of Oil Crops, Ministry of Agriculture, Wuhan, China; ^4^Key Laboratory of Plant Stress Biology, State Key Laboratory of Cotton Biology, School of Life Sciences, Henan University, Kaifeng, China; ^5^CAS Key Laboratory of Soybean Molecular Design Breeding, Northeast Institute of Geography and Agroecology, Chinese Academy of Sciences, Changchun, China

**Keywords:** high yield, florigen, *FT*, photosynthesis, soybean, vegetative growth

## Abstract

Crop yield has been maintaining its attraction for researchers because of the demand of global population growth. Mutation of flowering activators, such as florigen, increases plant biomass at the expense of later flowering, which prevents crop maturity in the field. As a result, it is difficult to apply flowering activators in agriculture production. Here, we developed a strategy to utilize florigen to significantly improve soybean yield in the field. Through the screening of transgenic lines of RNAi-silenced florigen homologs in soybean (*Glycine-max-Flowering Locus T Like*, *GmFTL*), we identified a line, *GmFTL*-RNAi#1, with minor changes in both *GmFTL* expression and flowering time but with notable increase in soybean yield. As expected, *GmFTL*-RNAi#1 matured normally in the field and exhibited markedly high yield over multiple locations and years, indicating that it is possible to reach a trade-off between flowering time and high yield through the fine-tuning expression of flowering activators. Further studies uncovered an unknown mechanism by which *GmFTL* negatively regulates photosynthesis, a substantial source of crop yield, demonstrating a novel function of florigen. Thus, because of the highly conserved functions of florigen in plants and the classical RNAi approach, the findings provide a promising strategy to harness early flowering genes to improve crop yield.

## Introduction

The global crop demand for human consumption and livestock feed is forecasted to increase by 110% from 2005 to 2050 (Tilman et al., 2011). However, advances in breeding, genomics, and transgenic technology are predicted to increase yield by up to 20% (Long et al., 2015). Therefore, there is an urgent need to develop innovative approaches to increase crop yields. Photosynthesis provides a substantial means to adjust crop yields (Ort et al., 2015). Regulation of photosynthesis happens at multiple levels (Long et al., 2006; Parry et al., 2007), which could serve as targets to enhance the efficiency of photosynthesis and, therefore, crop yields (Ort et al., 2015; South et al., 2019). Increasing photosynthetic efficiency would include improving the photosynthetic process through changing the structure and physiology of the chloroplast in multiple targets, therefore avoiding potentially harmful effects from the alteration of a single factor.

Unsurprisingly, flowering time is widely used as a selectable marker in high-yield plant breeding programs (Blumel et al., 2015). A long vegetative phase means later flowering and high yield because it provides numerous resources for increased yield. However, crops in the field may not mature normally before winter if the vegetative phase is too long. Therefore, balancing vegetative growth and reproductive growth will achieve high yield in a normal growth season. The transition from the vegetative to the reproductive phase is regulated by a complex genetic network. Plant monitors and integrates both the developmental and environmental signals to produce florigen (*Flowering Locus T*, *FT*) (Yoo et al., 2005; Corbesier et al., 2007; Tamaki et al., 2007; Andres and Coupland, 2012). The lower the florigen production, the later the flowering and the higher the yield (Andres and Coupland, 2012; Blumel et al., 2015; Cho et al., 2017). The *FT* dosage plays a key role in the yield of tomato (Krieger et al., 2010) and rice (Huang et al., 2016). However, utilizing flowering genes, such as the *FT* gene, to increase crop yield in the field remains unknown.

In addition to flowering control, *FT* homologs also contribute to the regulation of vegetative growth, namely, tuberization (Navarro et al., 2011), onion bulb formation (Lee et al., 2013), sugar beet growth (Pin et al., 2010), and seed dormancy (Chen and Penfield, 2018). However, the understanding of *FT* in regulating leaf growth is limited, even though it is reported that overexpression of *FT* leads to smaller leaves and reduced expression leads to increased leaf size in *Arabidopsis* (Teper-Bamnolker and Samach, 2005). There is no report showing the relationship between the *FT* gene and photosynthesis.

Here, we show that a decreased expression of the florigen gene significantly enhances chloroplast and leaf development, as well as photosynthetic efficiency, and therefore increases soybean yield. We further test the effect of silencing florigen on field soybean yield, and screen out the transgenic line *GmFTL*-RNAi line #1, which shows flowering time quite similar to that of wild-type plants and has significantly higher yield in the field. Therefore, we develop a smart strategy to utilize flowering activators for crop yield improvement.

## Results

### *GmFTL* Expression Is Negatively Correlated With Soybean Yield Under Controlled Conditions

*GmFTL* is a soybean homolog of *FT* (Kong et al., 2010; Fan et al., 2014; Nan et al., 2014; Guo et al., 2015), a key controller for flowering initiation (Yoo et al., 2005; Corbesier et al., 2007; Tamaki et al., 2007; Andres and Coupland, 2012). Among them, *GmFTL3* and *GmFTL4* are strong candidates of *FT* genes in soybean (Kong et al., 2010; Fan et al., 2014). We previously reported that silencing *GmFTL* genes delays flowering in soybean (*Glycine max* cv. Tianlong1) (Guo et al., 2015). To investigate if *GmFTL* genes were involved in yield production, we selected four *GmFTL*-RNAi transgenic lines (RNAi line #1, RNAi line #3, RNAi line #4, and RNAi line #5) (Guo et al., 2015) for further study under controlled conditions. First, the expression levels of two major *FT* genes, *FTL3* and *GmFTL4*, in soybean leaves (Kong et al., 2010; Fan et al., 2014; Nan et al., 2014; Guo et al., 2015) were evaluated in a growth room. As Supplementary Figure 1 shows, both genes displayed lower expression levels in transgenic lines. Among them, the abundance of *FTL3* and *GmFTL4* in either *GmFTL*-RNAi #4 or *GmFTL*-RNAi #5 was quite similar but was only one-tenth in wild-type plants, while *GmFTL*-RNAi#1 did not show a significant difference from wild-type plants. The levels of *GmFTL*3/4 in *GmFTL*-RNAi#3 plants were between those of the wild-type and *GmFTL*-RNAi#4/5 plants. The results suggest that the effect of *GmFTL*-RNAi was much stronger on *GmFTL*-RNAi#4 and #5. Therefore, we focused on *GmFTL*-RNAi#4 as the main material, also combined with other lines in some cases, in the sequential studies under controlled conditions.

Larger shoots and roots of RNAi line #4 were easily observed (Supplementary Figure 2). The RNAi lines were taller with more nodes, and this phenotype was negatively correlated with the expression levels of *GmFTL3* and *GmFTL4* (Supplementary Figure 3). Therefore, these results suggest that *GmFTL* functions in promoting vegetative growth. However, RNAi line #3 had significant effect on branching and increased the number of branches, while other transgenic lines did not cause much difference in branching phenotypes compared with wild-type plants (Supplementary Figure 3).

**FIGURE 1:**
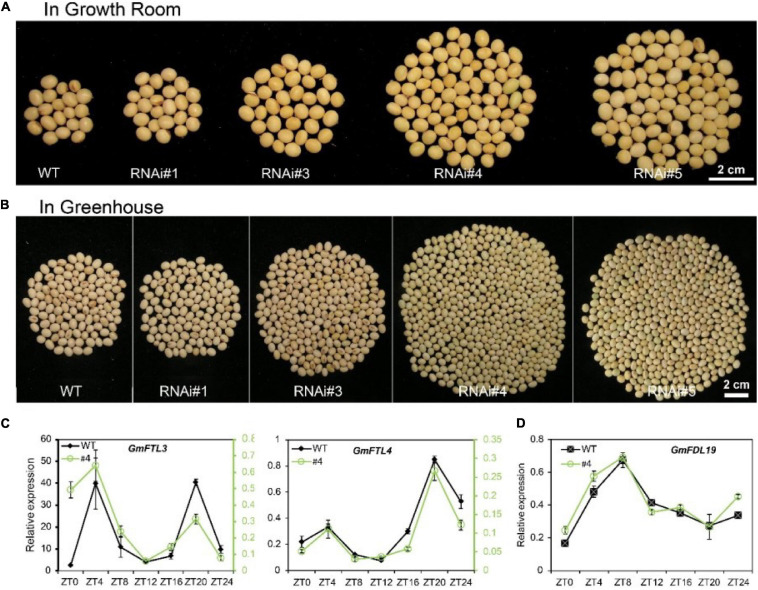
Reducing florigen expression increases soybean yield under controlled conditions. **(A,B)** Soybean yield per plant of different *GmFTL*-RNAi transgenic lines (#1, #3, #4, and #5) and WT in the **(A)** growth room and **(B)** greenhouse. Refer to Supplementary Figure 6 for statistical analysis of panels **(A,B)**. **(C)** Circadian expression of *GmFTL3* and *GmFTL4* under short day conditions (8-h light/16-h dark) detected by TagMan analysis. *GmUKN2* (Glyma06g04180) (Hu et al., 2009) was employed as a reference gene. For *GmFTL3* and *GmFTL4*, the data of wild-type plants (WT, black line) are presented on the left *Y*-axis, while the data of RNAi line #4 (#4, green line) are drawn on the right *Y*-axis. **(D)** The expression of *GmFDL19* under short day conditions (8-h light/16-h dark) was evaluated by RT-qPCR. *GmACT11* was employed as a reference gene.

There is no doubt, similar to other flowering plants, the flowering time and podding time are closely linked to the expression level of *FT* genes in soybean. Decreased *GmFTL3* and *GmFTL4* expression was attributed to late flowering (Supplementary Figure 4) and late podding (Supplementary Figure 5), suggesting a longer maturity phase for *GmFTL*-RNAi lines. Compared with wild-type plants, these *GmFTL*-RNAi lines displayed higher yield in both the growth room and greenhouse (Figures 1A,B and Supplementary Figure 6). RNAi line #1 also showed a slightly higher yield than wild-type plants, but this was not significant. The number of pods and seeds per plants likely contributed to the yield increase (Supplementary Figure 7) and such high yield did not occur at the cost of seed quality (Supplementary Figure 8). Obviously, higher intensity of light enhanced such yield-increasing effect, because the difference in yield between transgenic lines and wild-type plants was much greater in the greenhouse than in the growth room (Figures 1A,B, Supplementary Figure 6, and Supplementary Table 1). The length of light duration also had an obvious impact on yield, because the yield of WT and *GmFTL*-RNAi was higher in the greenhouse than in the growth room. In the growth room, the light/dark cycle is 8-h lighting from LEDs combined with 16-h dark; whereas in the greenhouse, the 24-h light/dark cycle has around 13-h lighting from sunlight with supplemental LEDs from 7:00 to 10:00 AM and 5:00 to 8:00 PM. These results suggest that the duration of illumination affected the growth period, and that longer illumination prolonged the growth period, resulting in higher yield (Supplementary Figure 6). These results are consistent with typical characteristics of short-day plants, such as soybean (Bernier and Perilleux, 2005). It is also observed that even though the expression level of *GmFTL3* and *GmFTL4* and the flowering time were quite similar between transgenic lines #4 and #5, the difference in the yield increase was significant (Supplementary Figures 1, 4, 6, 7), suggesting that a minor change in the *GmFTL* transcript level would lead to a major change in yield. It should be noticed that a higher yield in *GmFTL*-RNAi lines #3, #4, and #5 occurred at the expense of a longer growth period (Figure 1 and Supplementary Figures 4–6).

In *Arabidopsis* and rice, florigen interacts with a transcription factor FLOWERING LOCUS D (FD) (Taoka et al., 2011). We also found that GmFTL3 and GmFTL4 proteins interacted with GmFDL19 (Supplementary Figure 9), consistent with a previous report (Nan et al., 2014). No major perturbations in circadian expression of *GmFDL19* were observed in RNAi line #4 (Figure 1D). What is more, the circadian rhythm pattern of the *GmFTL3* and *GmFTL4* expression did not change (Figure 1C), which supports that the yield increase in *GmFTL*-RNAi lines results from the change in *GmFTL3* and *GmFTL4* genes at transcript abundancy.

Florigen produces in leaves and then is transported to the apexes to initiate flowers (Corbesier et al., 2007; Tamaki et al., 2007; Turck et al., 2008). *FD*, a functional partner of florigen, also has a potential function in leaves (Jang et al., 2017). Next, we confirmed that the effect of *GmFTL*-RNAi on yield was dominated by shoots or roots through a grafting approach experiment between *GmFTL*-RNAi line #4 and wild-type plants. The results showed that the composite plants with *GmFTL*-RNAi shoots as a scion flowered later and had more seeds and larger roots than that with wild-type shoots as a scion (Supplementary Figure 10). However, the composite plants with wild-type plants as a scion had little effect on the related phenotypes. The results indicate that the shoots dominate the yield, flowering time, and root growth in *GmFTL*-RNAi line #4 plants.

### Florigen Inhibits Leaf Growth

**FIGURE 2:**
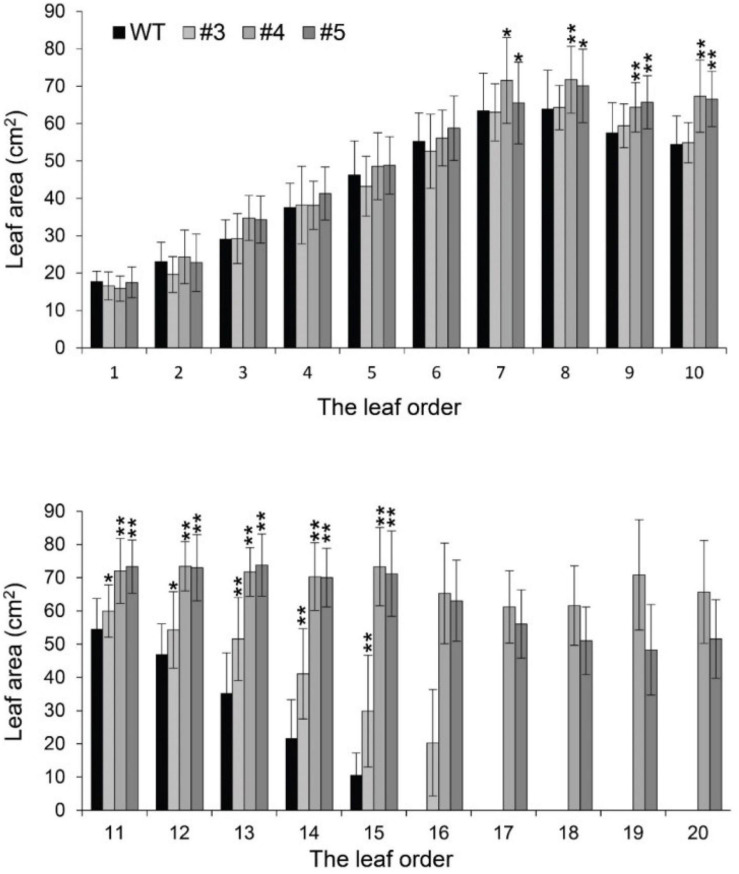
Reduction of *GmFTL* transcripts promotes trifoliolate leaf growth. The area of the new, fully opened trifoliolate leaves of wild-type plants and *GmFTL*-RNAi lines #3, #4, and #5 was analyzed. The leaf order was based on the developmental subsequence of trifoliolate leaves. * and ** indicate *P* < 0.05 and < 0.01, respectively. Student’s *t*-test, *n* = 7.

**FIGURE 3:**
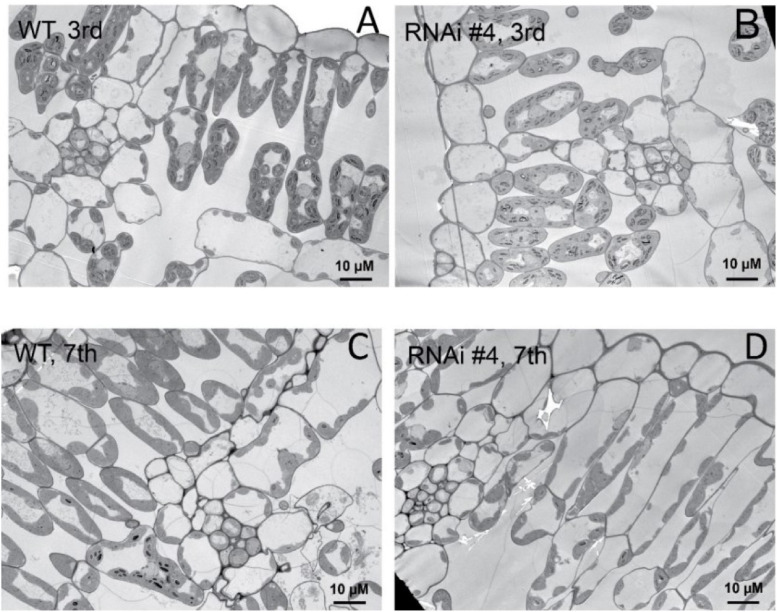
*GmFTL*-RNAi enhances leaf cell growth. Transmission electron micrographs of the new, fully opened third **(A,B)** and seventh **(C,D)** trifoliolate leaves of wild-type **(A,C)** or the *GmFTL*-RNAi line #4 plants **(B,D)** grown in greenhouses. The seventh trifoliolate leaves show larger and longer cells in *GmFTL*-RNAi lines compared with wild-type plants. Scale bar, 10 μm.

Beyond flowering regulation, florigen may be involved in many other development processes because it is expressed in many other tissues and organs besides leaf veins (Liu et al., 2014). A previous report proved that *FT* functions in leaf development (Teper-Bamnolker and Samach, 2005). We observed a visual phenotype that *GmFTL*-RNAi plants had greater number and sizes of leaves than wild-type plants (Figure 2 and Supplementary Figure 11). A lower expression level of *FT* gene enhanced leaf growth, especially that of later initiated leaves (produced after the seventh trifoliolate leaves), which had a much larger size than early initiated ones, suggesting that the role of *GmFTL* in leaf growth and initiation is in a developmental stage-dependent mode. We further investigated the leaf structure in leaf sections. Transmission electron microscopy clearly showed that there was no significant difference in cell size of the early initiated leaves (the third trifoliolate leaves) between wild-type and *GmFTL*-RNAi line #4 plants (Figures 3A,B). However, the cell size in the later initiated leaf (the seventh trifoliolate leaves) of *GmFTL*-RNAi line #4 was larger and longer (Figures 3C,D), suggesting that *GmFTL* was involved in leaf cell growth in a developmental stage-dependent mode in soybean. Previous studies have shown that florigen expresses increasingly according to developmental progress (Kardailsky et al., 1999; Krzymuski et al., 2015); and in that way, it is no surprising that the effect of *GmFTL*-RNAi is much obvious at late developmental stages. Taken together, the results suggest that *GmFTL* inhibits leaf growth and development in soybean.

### Florigen Negatively Controls Photosynthesis

**FIGURE 4:**
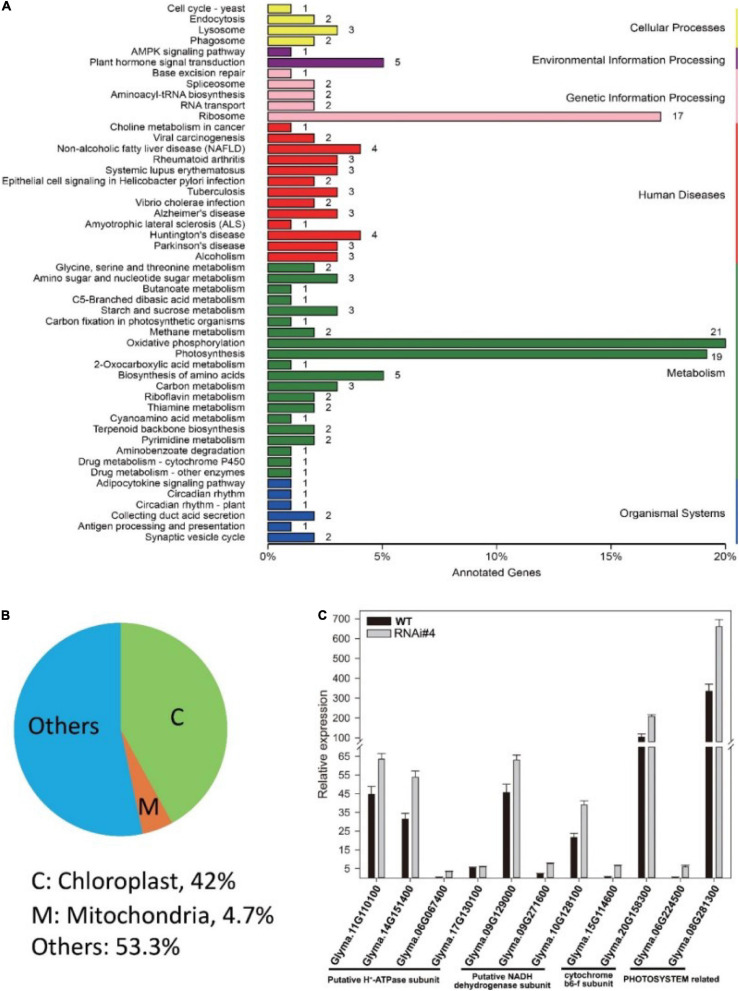
*GmFTL-*RNAi significantly impacts the expression of genes related to photosynthesis. Transcriptome analysis of the third trifoliolate leaves of *GmFTL-*RNAi line #4 and WT at ZT1. **(A)** Differential expression genes (DEG, fold changes ≥ 2 and false discovery ≤ 0.05) were enriched in the KEGG pathways “Photosynthesis,” “Oxidative phosphorylation,” and “Ribosome,” which are mainly related to energy metabolism. **(B)** Forty-two percent of DEGs coded proteins targeting the chloroplast. Refer to Supplementary Table 1 for the list of differential genes. **(C)** RT-qPCR verified the expression of 11 genes with more than two-fold changes detected by transcriptome analysis that are related to photosynthesis. The error bar indicates the standard deviation of three replicates.

Next, we tried to elucidate the mechanism of *GmFTL* expression in soybean yield through transcriptome analysis of the third trifoliolate leaves of *GmFTL*-RNAi line #4 and WT at Zeitgeber1 (ZT1). Unexpectedly, RNA-seq data showed that silencing *GmFTL* caused expression change in only a small portion of coding genes in the soybean genome (0.378%, 212 out of 56,044, *G. max* Wm82.a2.v1) (Supplementary Table 2, differential expression genes, fold changes ≥2 and false discovery ≤0.05). Among them, chloroplast-related genes were highlighted (Figures 4A,B), suggesting that the effect of *GmFTL*-RNAi on leaves is limited and specific. Then, we selected genes related to chloroplast functions to confirm RNA-seq data by RT-qPCR. These genes included putative H^+^-ATP subunits (Glyma.11G110100, Glyma.14G151400, Glyma.06G067400, and Glyma.17G130100), putative NADH dehydrogenase subunits (Glyma.09G129000, Glyma.09G271600, and Glyma.10G128100), cytochrome b6f subunits (Glyma.15G114600 and Glyma.20G158300), and photosystem related genes (Glyma.06G224500 and Glyma.08G281300). The RT-qPCR results were in agreement with the RNA-seq data (Figure 4C). These data indicate that *GmFTL* may have a specific effect on chloroplast development and functions in soybean plants.

**FIGURE 5:**
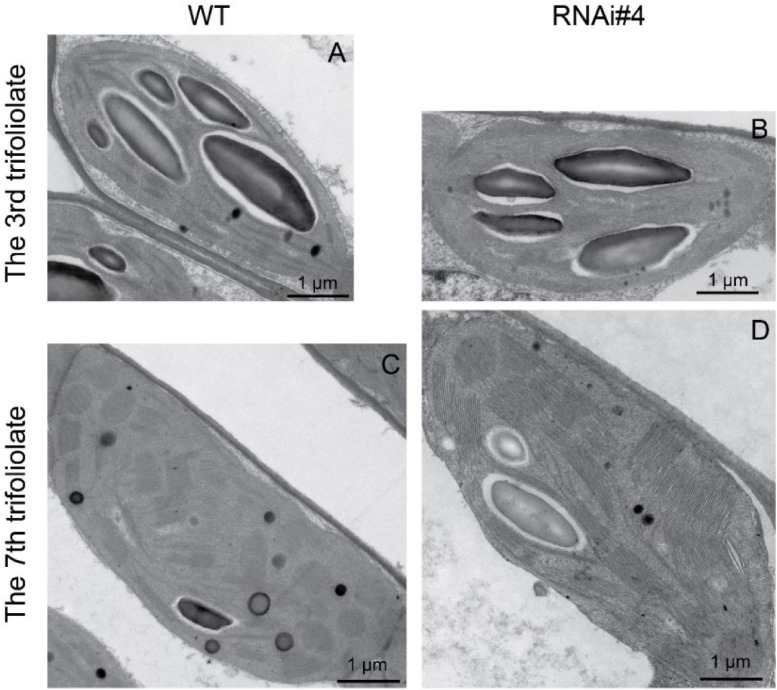
*GmFTL*-RNAi enhances chloroplast development. The representative transmission electron micrographs of chloroplasts in the third **(A,B)** and seventh trifoliolate leaves **(C,D)** from wild-type plants **(A,C)** or *GmFTL* RNAi line #4 **(B,D)**. Scale bar, 1 μm.

The structure of chloroplasts reflects the function of photosynthesis, and more thylakoid membranes and rich grana contribute to higher efficiency of photosynthesis and the formation of photosynthetic products (Jensen and Leister, 2014; Pribil et al., 2014; Kirchhoff, 2018). Therefore, we checked the characteristics of the chloroplast structure by transmission electron microscopy. The results showed that the chloroplasts of the *GmFTL*-RNAi#4 leaves exhibited much more complicated structures with more and wider thylakoid membranes and richer grana than those in wild-type plants, regardless of the early or later initiated leaves (Figure 5).

**FIGURE 6:**
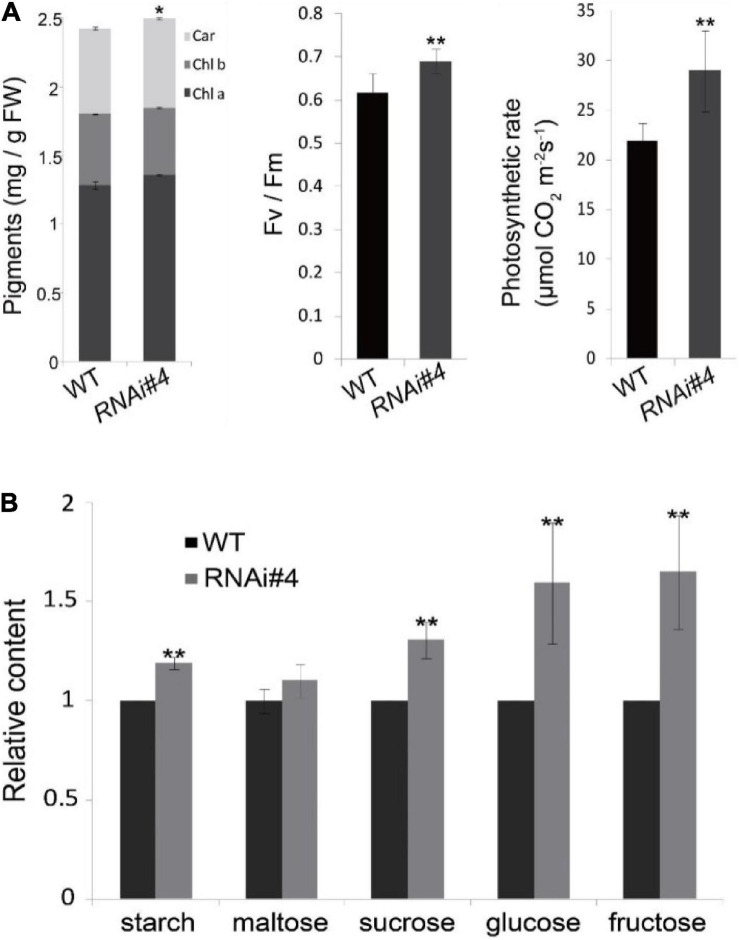
*GmFTL*-RNAi enhances photosynthesis in soybean. **(A)** The contents of photosynthetic pigments, chlorophylls a and b, and carotenoids **(left)**, the maximum quantum yield **(middle)**, and the net photosynthesis **(right)** of the third leaves in trifoliolate leaves of wild-type plants and the *GmFTL* RNAi line #4 in a growth room. **(B)** The third trifoliolate leaves of *GmFTL* RNAi line accumulated more sugars compared with wild-type plants. *n* = 10 plants. * and ** indicate *p* < 0.05 and < 0.01, respectively. *n* = 10 plants.

All the properties above may confer higher photosynthetic efficiency of *GmFTL*-RNAi line leaves. Then, we analyzed the physiological and biochemical characteristics of the leaves. The biochemical assay indicated that the *GmFTL*-RNAi line #4 leaves were enriched in photosynthetic pigments, had higher maximum quantum efficiency and photosynthetic rates (Figure 6A), and accumulated more photosynthetic assimilates such as starch, maltose, sucrose, glucose, and fructose (Figure 6B). Thus, the transgenic *GmFTL*-RNAi plants had higher photosynthetic efficiency than the wild-type plants, and *GmFTL* negatively regulated photosynthesis.

### A Slightly Decrease in Florigen Enhances Soybean Yield in the Field

**FIGURE 7:**
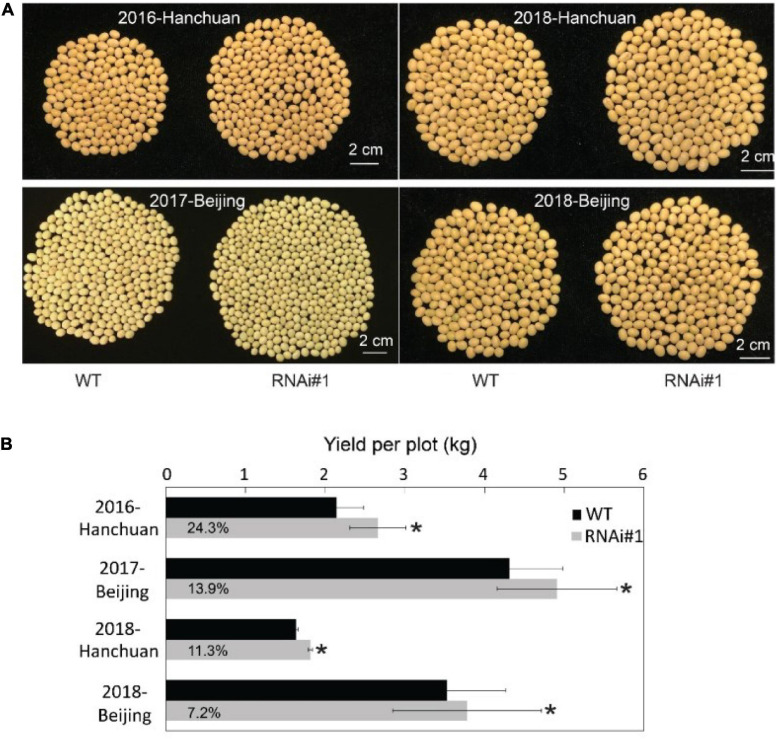
Slightly reducing *GmFTL* expression increases soybean yield in the field. **(A)** *GmFTL*-RNAi line #1 was subjected to a field yield investigation in Hanchuan and Beijing from 2016 to 2018. The photos show the total number of seeds from a single representative plant. **(B)** Statistical assay of yield per plot (kg) in the field investigation. The digits in the gray bars indicate the percentage of yield increase in *GmFTL*-RNAi#1 over wild-type plants. *Indicates significant differences at *P* < 0.05 based on Student’s *t*-test. *n* = 5.

All of the presented data above were from samples under controlled conditions. Then, we determined what would happen when these transgenic lines grew under natural field conditions. First, we planted *GmFTL*-RNAi lines #1, #3, and #4 in the field. Only *GmFTL*-RNAi line #1 matured naturally; and *GmFTL*-RNAi lines #3 and #4 did not mature before winter because they flowered too late. Therefore, we focused on *GmFTL*-RNAi line #1 for field experiments in two different environments (Beijing and Hanchuan) across years (from 2016 to 2018). In the field, there was not much difference between *GmFTL*-RNAi line #1 and wild-type plants during the vegetative stage (Supplementary Figure 12). However, at the fully mature stage, *GmFTL*-RNAi line #1 had more pods than wild-type plants (Supplementary Figure 13). Except for some cases of failure due to diseases, the yield increase in *GmFTL*-RNAi line #1 ranged from 7.2 to 24.2% (Figure 7). We also found that the yield increase in the original habitat (Hanchuan, N30°22′, E113°22′, where the parent line of *GmFTL*-RNAi line #1 originates from) was higher than that in the other environment (Beijing, N39°58′, E116°20′). We postulated that the altitude of growth regions restricts *GmFTL* functions, because soybean is an obligate short day and photoperiod-sensitive plant (Borthwick and Parker, 1938; Nanda and Hamner, 1959; Zhang et al., 2001). So, *GmFTL*-RNAi line #1 had at least an 11% yield increase compared with its parent in the original parent habitat (Figure 7B, 2018-Hanchuan). The result indicates that *GmFTL*-RNAi line #1 may be a high-yield elite candidate in the field, and that RNAi of florigen is a potential strategy to improve soybean yield.

## Discussion

Vegetative growth is a double-edged sword for reproductive growth: it is the foundation of reproductive growth, but an extended period of vegetative growth inhibits reproductive growth. Plants have evolved multiple strategies to balance these two essential processes, so that they can flower at an appropriate time and set enough healthy seeds to survive and prosper. Florigen, as a central activator of flowering time, may play a key role in balancing vegetative growth and reproductive growth. As a result of that, florigen is difficult to be applied in practical production, because its knockout leads to late flowering and failure of normal maturation in the field, whereas its overexpression results in early flowering and lower yield. The florigen dosage has a dominant role in regulating crop yield (Krieger et al., 2010; Huang et al., 2016). However, how to utilize florigen to increase crop yield in the field remains unknown. In addition, there is no report showing that florigen is related to photosynthesis.

### Florigen Inhibits Photosynthesis and Yield Production

We demonstrate that the knockdown of florigen significantly enhances leaf growth and chloroplast development. *GmFTL*-RNAi lines have three typical advantages conferring high photosynthesis compared with wild-type plants: (1) more and larger leaves (Figure 2) with longer leaf cells (Figure 3); (2) chloroplasts with much more complicated membranes and grana (Figure 5); and (3) high amount of photosynthetic pigments (Figure 6). Such functions of *GmFTL*-RNAi are likely specific, because only few genes (212 out of 56,044 genes in the soybean genome, Supplementary Table 2) showed significant changes in expression, and 42% of these genes code proteins targeting chloroplasts (Figure 4). Therefore, it is no surprising that *GmFTL*-RNAi lines have more efficient photosynthesis, and they accumulate more photosynthetic products (Figure 6) and, finally, produce more seeds independent of environmental conditions (Figures 1, 7). However, growth conditions impact the function of *GmFTL*-RNAi, and higher intensity and longer duration of illumination enhance the effect (Figures 1, 7 and Supplementary Table 1). The latitude of growth region is also linked to the function of *GmFTL*-RNAi; that is, the original habitat benefits the effect of *GmFTL*-RNAi (Figure 7), which can be tracked to native the habitat of a variety because soybean is an obligate short-day plant.

The *FT* gene is highly conserved in sequences and functions across the plant kingdom and plays multiple roles in many important processes beyond flowering time control (Pin et al., 2010; Navarro et al., 2011; Lee et al., 2013; Chen and Penfield, 2018). However, there is no study showing *FT* is related to photosynthesis, even though one study claims that *FT* has non-negligible functions in leaf growth in *Arabidopsis* (Teper-Bamnolker and Samach, 2005). This study bridges the gap between florigen and both photosynthesis and vegetative growth, which provides a cue to elucidate the mechanism of balancing vegetative growth and reproductive growth. It is interesting to identify the direct targets of *GmFTL* in leaves to establish a network coordinating vegetative growth and reproductive growth mediated by *GmFTL*.

### Fine-Tuning Florigen Expression Is a Promising Strategy for High Yield

Crop yield attracts researchers because of the increasing global population. Numerous approaches have been employed to improve crop yield, such as manipulating the expression of homologous (Preuss et al., 2012; Do et al., 2016; Ge et al., 2016) or heterologous genes in soybean (Li et al., 2013; Waltz, 2014; Kohler et al., 2016). Some strategies show high yield only under stress conditions (Li et al., 2013; Waltz, 2014; Do et al., 2016), whereas field trials have not been conducted (Waltz, 2014; Do et al., 2016; Ge et al., 2016). The approach of the authors shows soybean grain yield produced in 3 years in two field trials. Evaluation of the flowering time and yield of *GmFTL*-RNAi #1, compared with wild-type plants in the field or *GmFTL*-RNAi #4 compared with *GmFTL*-RNAi #5 under control conditions (Supplementary Figures 1, 6), revealed the importance of *GmFTL* level; that is, the yield difference would be significant even though flowering time is quite similar. Therefore, a slight reduction in the *FT* expression increases soybean yield in the field (Figure 7) but not at the expense of late flowering or seed quality (Supplementary Figures 1, 8), important traits for agricultural application (Blumel et al., 2015). The yield increase in the original habitat (Hanchuan) was 11–24% (Figure 7B). It is possible to screen more transgenic *GmFTL*-RNAi lines with different florigen levels to obtain a much higher yield in the field.

Different environmental conditions affect the yield of *GmFTL*-RNAi lines (Figures 1, 7), indicating that environmental cues, such as light characteristics, participate in the network of *GmFTL*-RNAi regulation of yield (or photosynthesis). Therefore, it is interesting to develop a new strategy for modifying the light signaling pathway to increase soybean yield.

In this study, we employed one key gene, florigen, and common biotechnology, RNAi. Florigen is a highly conserved gene in the plant kingdom that enhances plant flowering (Wickland and Hanzawa, 2015). RNAi is a classical approach to reducing gene expression. To confirm such results, we also analyzed the *FT*-RNAi effect on leaf development in *Arabidopsis thaliana*. As expected, the number of rosette leaves was inversely proportional to the abundance of *FT* mRNA (Supplementary Figure 14), and *FT*-RNAi increased the biomass and the size of leaves of *Arabidopsis*. Therefore, we predict that the strategy we have shown here can be widely applied to different crops to increase their yields in the field. Beyond florigen, many genes are involved in the regulation of flowering time, and similar challenges will be met when these flowering genes are applied in agricultural production. The strategy provides a smart example for the application of such genes.

## Materials and Methods

### Plant Materials and Growth Conditions

*GmFTL*-RNAi plants were previously generated in the soybean [*G. max* (L.) Mer.] cultivar Tianlong1 in the laboratory of the authors (Guo et al., 2015). All the RNAi lines used here are independent, homozygous, transgenic ones. The wild-type soybean control for all of the experiments is cultivar Tianlong1, which originates in Hanchuan, China. All of the plants were grown under controlled temperature and photoperiod growth rooms, greenhouses, and/or the field. The light conditions in the plant growth rooms are short day conditions (8 h light/16 h dark) from a LED light source (GreenPower LED top lighting, Philips Horticulture LED).^1^ Natural light and temperature conditions were used in the greenhouse, with supplemental LED light from 7:00 to 10:00 AM and 5:00 to 8:00 PM. The light spectrum and intensity in the growth room and greenhouse are listed in Supplementary Table 1.

Soybean field (plot) experiments were carried out in Hanchuan (N30°22′, E113°22′) and Beijing (N39°58′, E116°20′) from 2016 to 2018. Spring sown soybeans were planted on 23rd April 2016 for Hanchuan, and May 15 or June 1, 2017 for Beijing. The plot area was 300 cm × 225 cm with 45-cm row-spacing and 20-cm plant-spacing in Hanchuan or 600 cm × 300 cm with 60-cm row-spacing and 30 cm plant-spacing in Beijing. Agronomic characters were determined at maturity. Individual plants from each plot were subjected to statistical analysis.

### RNA Extraction and Expression Analysis

Total RNA of leaves was extracted using an EasyPure^®^ RNA Kit (ER101-01, TransGen Biotech, Beijing, China). The quantity was measured with Nanodrop 2000C (Thermo Fisher Scientific, Waltham, MA, United States). For SYBR detection of RT-qPCR products, 500–1,000 ng of the total RNA were used for reverse transcription (KR106-02, TIANGEN, Sichuan, China). SYBR Premix Ex-Taq (Perfect Real Time; TaKaRa, Tokyo, Japan) was used for the RT-qPCR assays. For TaqMan^TM^ analysis, about 1 μg of total RNA was used for reverse transcription (KR106-02, TIANGEN, Sichuan, China), and TaqMan Gene Expression Master Mix (No.4369016, Thermo Fisher Scientific, Waltham, MA, United States) was used for the assays. RT-qPCR was conducted using StepOne Plus (ABI, United States). The reference gene *GmACT11* (Glyma.18G290800) or *GmUKN2* (Glyma06g04180) (Hu et al., 2009) was used as internal control. Sequences of the primers are listed in Supplementary Table 3. The 2^–Δ*CT*^ method was used to calculate the relative expression levels based on three technical replicates.

### Bimolecular Fluorescence Complementation

The pEarlyGate202-*GmFDL19-cYFP* and pEarlyGate202-*GmFTL3-nYFP* or pEarlyGate201-*GmFTL4-nYFP* binary vectors were transiently expressed using *Agrobacterium tumefaciens*. Both recombinant *Agrobacterium* cells were co-infiltrated into *Nicotiana benthamiana* leaves. Empty vectors were used as negative controls, and AtAHL22-RFP was used as a nuclear marker (Xiao et al., 2009). *N. benthamiana* was grown under long-day (16-h light:8-h dark) conditions at 22°C for at least 48-h post infiltration. Leaves were observed under a confocal microscope (Zeiss LSM700, Jena, Germany).

### Chloroplast Analysis and Chlorophyll Measurements

The new, fully opened third and seventh trifoliolate leaves of wild-type plants Tianlong1 and *GmFTL*-RNAi line #4 soybean plants in the greenhouse were harvested for measurements. Perpendicular transverse sections of the middle leaflets of trifoliolate leaves were prepared by the Transmission-Electron-Microscope and Mass-Spectrometry Platform of Institute of Agricultural Products Processing, Chinese Academy of Agricultural Sciences (Beijing, China). The photographs were obtained using a transmission electron microscope (H-7500, Hitachi, Tokyo, Japan).

For chlorophyll content analysis, we punched 20 fresh sections (*d* = 6 mm) from five individual leaves of each sample using a hole puncher. The samples were immersed in 25 mL of 80% acetone and stored at room temperature for 5 days in the dark. Then, the absorbance of 1 ml of the supernatants was measured at 663 and 645 nm. The concentration of chlorophylls a and b (Chl a and Chl b) and carotenoids was calculated using the following formulas:

Chla=(11.24A-6632.04A)645×V/W

Chlb=(20.13A-6454.19A)663×V/W

Carotenoids=((1000A-4701.9Chla-63.14Chlb)/214)×V/W

(*V* is the volume of acetone, and *W* is the fresh weight of the sample)

### Photosynthetic Rate and Chlorophyll Fluorescence Analyses

The plants were grown under SD conditions (growth room), and the third trifoliolate leaves (*n* = 10) of wild-type plants Tianlong1 and *GmFTL*-RNAi line #4 were selected to measure the photosynthetic rates at days 28–35 after sowing following the instructions of the manufacturer (LI-6400 V4.0.1, LI-COR, Lincoln, NE, United States). Fv/Fm was measured using an IMAGING-PAM M series Chlorophyll Fluorometer with the MAXI version (Heinz Walz, Effeltrich, Germany). The plants were placed in the dark for 30 min before measurements were taken.

### Measurement of the Sugar Content in Leaves

Soybean Tianlong1 and *GmFTL*-RNAi line 4 seeds were sown in a growth room. The third new, fully opened trifoliolate leaves were harvested at ZT1 for the measurement of sugar content (*n* = 5). For the extraction of soluble sugars and starch, about 100 mg of leaf samples was homogenized in 5 ml of 80% (v/v) ethanol in a 1.5-ml tube and incubated at 70°C for 90 min. Following centrifugation at 16,000 × *g* for 5 min, the supernatant was transferred to a new 1.5-ml tube. The pellet was rinsed twice with 2 ml of 80% ethanol and removed. Any remaining solvent was evaporated at room temperature using a vacuum. The residue was resuspended in.3 ml of distilled, sterile water, and this represented the soluble carbohydrate fraction. The remaining pellet containing insoluble carbohydrates, such as starch, was homogenized in 2 ml of 2 N KOH, and the suspension was incubated at 95°C for 1 h to dissolve the starch. Following the addition of.035 ml of 1 N acetic acid and centrifugation for 5 min at 16,000 × *g*, the supernatant was used for starch quantification. Detailed procedures were followed according to the instructions of the manufacturer; starch (No. 1013910603), maltose/sucrose/D-glucose (No. 11113950035) and D-glucose/D-fructose kits (No. 10139106035) (R-Biopharm, Pfungstadt, Germany).

### Measurement of Major Agronomic Traits

The flowering time was determined by the emergence of the first flower on the main stem of soybean plants. The podding time was determined as the time when the first pod on the main stem of the soybean plants was 2 cm in length. Measurements of plant height, branch number, node number, pod number and seed number per plant were performed at full plant maturity. For experiments in both the greenhouse and growth rooms, more than 10 individual plants of each line were sampled for analysis of all traits. For analysis of seed quality traits, 100 plump seeds of wild-type plants and transgenic lines were used. The content of proteins, oils, and moisture was determined using a near-infrared spectrometer (Brooke Technology Co., Ltd., Beijing, China) with the pre-stored soybean protein and oil model in the instrument. The operation was performed according to the instructions of the manufacturer.

### Transcriptome Analysis

The new, fully opened third trifoliolate leaves from wild-type plants or *GmFTL*-RNAi line #4 were sampled ZT1. Total RNA was extracted using an EasyPure^®^ RNA Kit (ER101-01, TransGen Biotech, Beijing, China). RNA sequencing with an Illumina HiSeq instrument and data analysis were performed by Biomarker Technologies (Beijing, China). Illumina sequencing reads were mapped to reference genome *G. max* Wm82.a2.v1.^2^ Transcriptome data were deposited to Genome Sequence Archive (GSA)^3^ with an accession number of CRA004267).

### Statistical Analysis

All experiments in this study were carried out with at least three replicates, all of which showed similar results. The figures showed only a representative result. Data in all bar graphs represent the mean ± SD. All statistical analyses were performed using the SPSS software package. Asterisks indicate significant difference based on a Student’s *t*-test (^∗∗^*P* < 0.01, ^∗^*P* < 0.05).

### Gene Accession Numbers

Sequence data for this article can be found in the Phytozome: *GmFTL3* (Glyma16g26660), *GmFTL4* (Glyma16g04830), *GmFDL19* (Glyma.19G122800), *GmACT11* (Glyma.18G290800), *GmUKN2* (Glyma06g04180), putative H^+^-ATP subunits (Glyma.11G110100, Glyma.14G151400, Glyma.06G067400, and Glyma.17G130100), putative NADH dehydrogenase subunits (Glyma.09G129000, Glyma.09G271600, and Glyma.10G128100), cytochrome b6f subunits (Glyma.15G114600 and Glyma.20G158300), photosystem related genes (Glyma.06G224500 and Glyma.08G281300), and *AtFT* (At1g65480).

## Data Availability Statement

The original contributions presented in the study are publicly available. This data can be found here: https://ngdc.cncb.ac.cn/gsa/browse/CRA004267.

## Author Contributions

Y-FF, X-MZ, and XF: conceptualization. KX, Y-FF, X-MZ, CZ, and HC: methodology. KX, X-MZ, HC, JZ, CZ, ZC, PH, and YM: investigation. KX, X-MZ, and Y-FF: formal analysis and validation. KX and Y-FF: visualization. Y-FF, KX, and XF: writing. XF, X-MZ, and Y-FF: funding acquisition. Y-FF and X-MZ: project administration. XZ, Y-FF, and YM: resources. Y-FF: supervision. All authors contributed to the article and approved the submitted version.

## Conflict of Interest

The authors declare that the research was conducted in the absence of any commercial or financial relationships that could be construed as a potential conflict of interest.

## Publisher’s Note

All claims expressed in this article are solely those of the authors and do not necessarily represent those of their affiliated organizations, or those of the publisher, the editors and the reviewers. Any product that may be evaluated in this article, or claim that may be made by its manufacturer, is not guaranteed or endorsed by the publisher.

## Footnotes

1
http://www.lighting.philips.com.cn/products/horticulture

2
https://phytozome.jgi.doe.gov/pz/portal.htm

3
https://ngdc.cncb.ac.cn/gsub/

## Supplementary Material

Supplementary Figure 1 *GmFTL*-RNAi reduces the mRNA abundance of *GmFTL3* and *GmFTL4* in different transgenic lines. WT and *GmFTL*-RNAi lines #1, #3, #4, and #5 grew in growth room, and the first trifoliolate leaves were harvested to investigate gene expression at ZT4 by RT-qPCR. *GmACT11* was used as a reference gene. Among these transgenic lines, line #1 shows slight change in *GmFTL3* and *GmFTL4* expressions. Error bars indicate the standard deviation of the mean of three replicates. An asterisk indicates significant difference compared with wild-type plant (^∗∗^, *P* < 0.01. Student’s *t*-test, *n* ≥ 5 plants).

Supplementary Figure 2 Reducing *GmFTL* expression enhances the growth of **(A)** shoots and **(B)** roots in soybean. WT and *GmFTL*-RNAi line #4 grown in greenhouse. Photos were taken at maturity of wild-type plants.

Supplementary Figure 3 Reducing *GmFTL* expression enhances the growth of **(A,B)** stems but not **(C)** branching. WT and *GmFTL*-RNAi line #4 grown in greenhouse. Agricultural traits were analyzed at maturity. An asterisk indicates significant difference compared with wild-type plants (^∗∗^*P* < 0.01. Student’s *t*-test, *n* ≥ 20 plants).

Supplementary Figure 4 Reducing *GmFTL* expression delays soybean flowering. WT and *GmFTL*-RNAi lines #1, #3, #4, and #5 grown in greenhouse. An asterisk indicates significant difference compared with wild-type plants (^∗∗^*P* < 0.01. Student’s *t*-test, *n* ≥ 20 plants).

Supplementary Figure 5 Reducing *GmFTL* expression delays soybean podding time. WT and *GmFTL*-RNAi lines #1, #3, #4, and #5 grown in greenhouse. An asterisk indicates significant difference compared with wild-type plants (^∗∗^*P* < 0.01. Student’s *t*-test, *n* ≥ 20 plants).

Supplementary Figure 6 Reducing *GmFTL* expression increases soybean yield. WT and *GmFTL*-RNAi lines #1, #3, #4, and #5 grown in growth room and greenhouse. This figure is supporting data for Figure 1. An asterisk indicates significant difference compared with wild-type plants (^∗∗^*P* < 0.01. Student’s *t*-test, *n* ≥ 10 plants).

Supplementary Figure 7 Reducing *GmFTL* expression increases the number of pods and seeds per plant. WT and *GmFTL*-RNAi lines #1, #3, #4, and #5 grown in greenhouse. An asterisk indicates significant difference compared with wild-type plants (^∗∗^*P* < 0.01. Student’s *t*-test, *n* ≥ 20 plants).

Supplementary Figure 8 Reducing *GmFTL* expression does not alter the quality of soybean seeds. *GmFTL*-RNAi lines #4 and #5 grown in greenhouse have similar contents of **(A)** proteins, **(B)** oils, and **(C)** water as wild-type seeds.

Supplementary Figure 9 Both GmFTL3 and GmFTL4 proteins interact with GmFDL19 in the nucleus. *Agrobacterium tumefaciens* harboring pEarlyGate201-*GmFTL3:nYFP*, or pEarlyGate201-*GmFTL4:nYFP*, pEarlyGate202-*GmFDL19:cYFP* vectors were co-infiltrated into *Nicotiana benthamiana* leaves, respectively. Then, plants were incubated for 48 h and subsequently observed by bimolecular fluorescence complementation (BiFC) under a confocal microscope. AHL22-RFP was used as a nucleus marker protein. *GmFTL3:nYFP* and cYFP, *GmFTL4:nYFP* and cYFP, and nYFP and *GmFDL19:cYFP* were used as controls.

Supplementary Figure 10 Grafting assay. **(A)** Photos of different graft combinations and ungrafted control plants at maturity. For each graft combination, the genotypes of scion (above) and stock (below) were separated by a red line. **(B)** Flowering time and **(C)** seed number per plant for different graft combinations. Statistical significance at the 0.01 level was determined by Duncan’s multiple range test, *n* ≥ 3 plants.

Supplementary Figure 11 Reduction of *GmFTL* transcripts promotes growth of trifoliolate leaves. This photo shows morphology of trifoliolate leaves of *GmFTL*-RNAi #3, #4, and #5 grown in greenhouse. The leaf order is according to the developmental subsequence of trifoliolate leaves. This figure is a supporting data for Figure 4.

Supplementary Figure 12 *GmFTL*-RNAi line #1 does not show significant difference at the vegetative stage compared with wild-type plants. This photo shows plants of *GmFTL*-RNAi #1 grown in the field in Hanchuan, the original location of wild-type plants, in 2018. The photo indicates there is no difference between *GmFTL*-RNAi #1 **(right)** and wild-type **(left)** plants. Seeds were sown on April 20 and photos were taken on June 5.

Supplementary Figure 13 *GmFTL*-RNAi line #1 shows significant difference at the reproductive stage from wild-type plants. This photo shows dry plants at maturity of *GmFTL*-RNAi #1 and wild-type plants grown in the field in Hanchuan, the original location of wild-type plants, in 2018. The photo displays that *GmFTL*-RNAi #1 plants (low row) are of bigger stature and have more pods than wild-type plants (up row).

Supplementary Figure 14 The expression level of florigen is positively related to biomass and leaf size in *Arabidopsis*. *AtFT*-RNAi lines were produced by introducing an RNAi fragment of the *Arabidopsis* florigen *FT* gene (At1g65480) into ecotype Columbia-0, and homozygous transgenic lines were used in all experiments. *ft-10* is a null mutant of the florigen *AtFT* gene. *Arabidopsis* plants (wild-type, *AtFT*-RNAi lines, and *ft-10* mutants) grew under long day conditions (growth room). Photos of **(A)** rosettes and **(B)** leaves were taken at flowering. The *Arabidopsis* florigen *FT* gene expression was determined by RT-qPCR at day 14 after germination **(C)**. The flowering time is negatively correlated to flowering time **(C)** under long day conditions.

Supplementary Table 1 Light information in growth room and greenhouse.

Supplementary Table 2 Transcriptome analysis of GmFTL-RNAi#4 leaves at ZT1.

Supplementary Table 3 List of oligonucleotide and primer sequences used in this study.
