# Supplementary material for: Fine-Tuning Florigen Increases Field Yield Through Improving Photosynthesis in Soybean
Source: Front Plant Sci. 2021 Aug 16;12:710754. doi: 10.3389/fpls.2021.710754 (PMC8415793; doi:10.3389/fpls.2021.710754)
Supplement: Supplementary Figure 1 — GmFTL-RNAi reduces the mRNA abundance of GmFTL3 and GmFTL4 in different transgenic lines. WT and GmFTL-RNAi lines #1, #3, #4, and #5 grew in growth room, and the first trifoliolate leaves were harvested to investigate gene expression at ZT4 by RT-qPCR. GmACT11 was used as a reference gene. Among these transgenic lines, line #1 shows slight change in GmFTL3 and GmFTL4 expressions. Error bars indicate the standard deviation of the mean of three replicates. An asterisk indicates significant difference compared with wild-type plant (∗∗, P < 0.01. Student’s t-test, n ≥ 5 plants). [file Data_Sheet_1.zip › Supplementary Figures S1-S14.PDF]

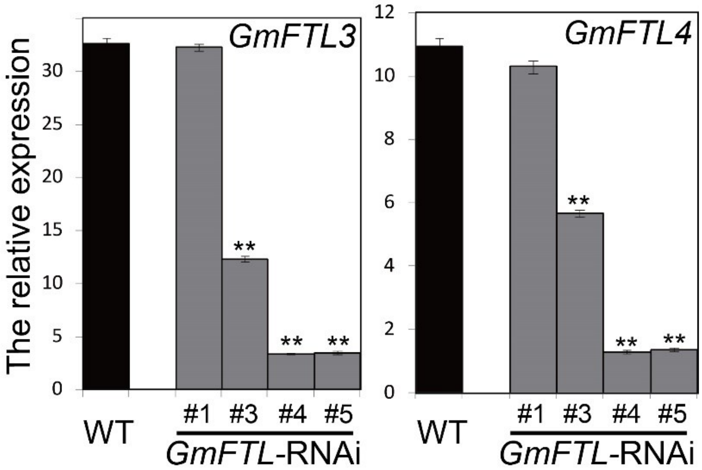

Figure S1 *GmFTL*-RNAi reduces the mRNA abundance of *GmFTL3* and *GmFTL4* in different transgenic lines. WT and *GmFTL*-RNAi line #1, #3, #4, and #5 grew in growth room and the first trifoliolate leaves were harvested to investigate gene expression at ZT4 by RT-qPCR. *GmACT11* was used as a reference gene. Among these transgenic lines, line #1 shows slightly change of *GmFTL3* and *GmFTL4* expressions. Error bars indicate the standard deviation of the mean of three replicates. An asterisk indicates significantly difference compared to wild type (**, *P* < 0.01. Student's *t*-test, n ≥ 5 plants).

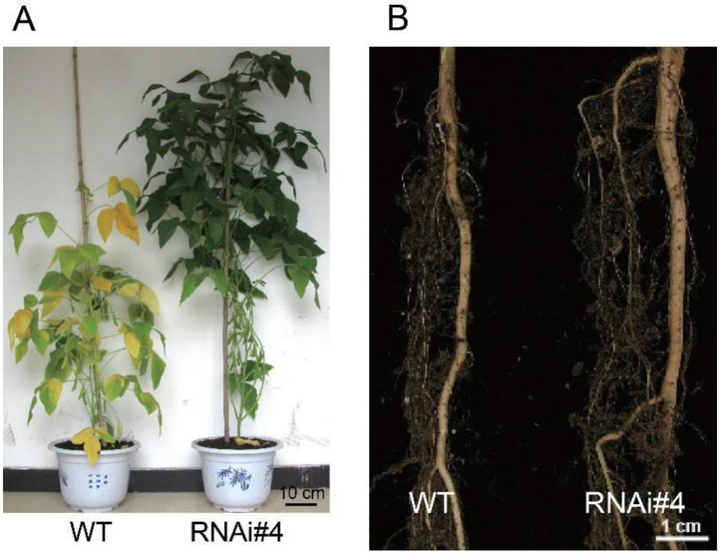

Figure S2 Reducing *GmFTL* expression enhances the growth of shoots (A) and roots (B) in soybean. WT and *GmFTL*-RNAi line #4 grew in greenhouse. Photos were taken at maturity of wild type plants.

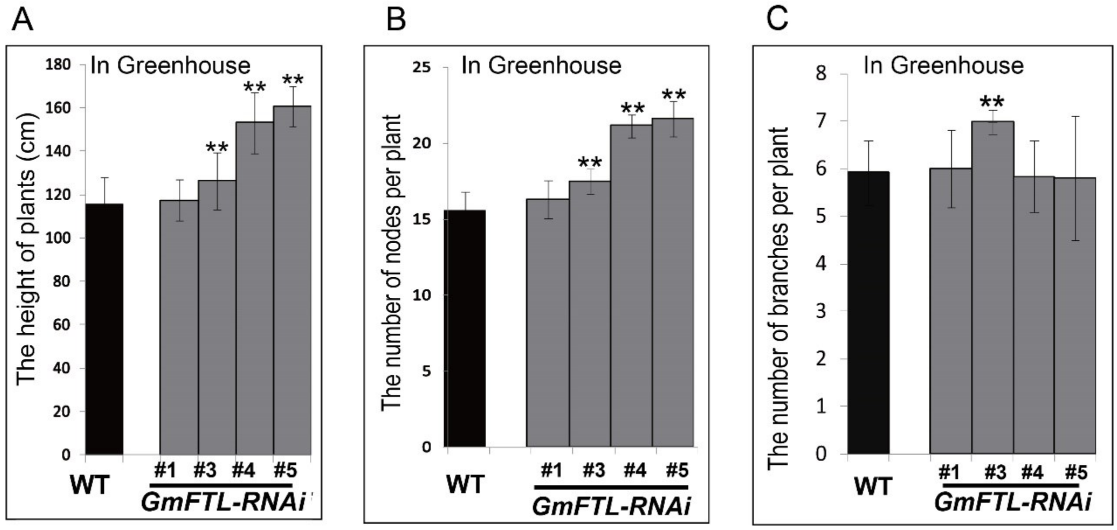

Figure S3 Reducing *GmFTL* expression enhances the growth of stems (A and B), but not branching (C). WT and *GmFTL*-RNAi line #4 grew in greenhouse. Agricultural traits were analyzed at maturity. An asterisk indicates significantly difference compared to wild type (**, $P < 0.01$. Student's *t*-test, n ≥ 20 plants).

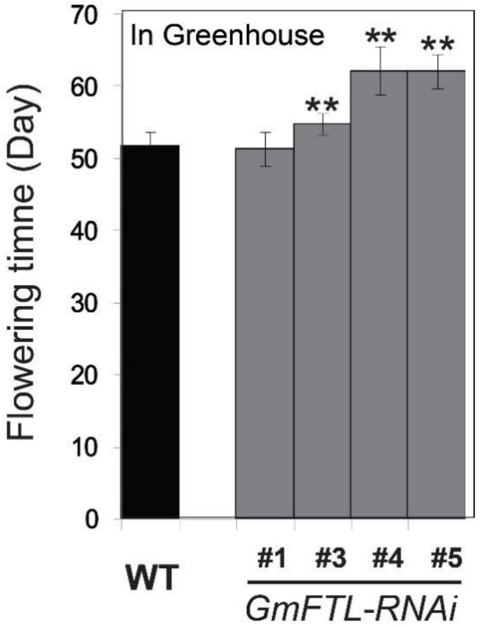

Figure S4 Reducing *GmFTL* expression delays soybean flowering. WT and *GmFTL*-RNAi line #1, #3, #4, and #5 grew in greenhouse. An asterisk indicates significantly difference compared to wild type (**, $P < 0.01$. Student's *t*-test, n ≥ 20 plants).

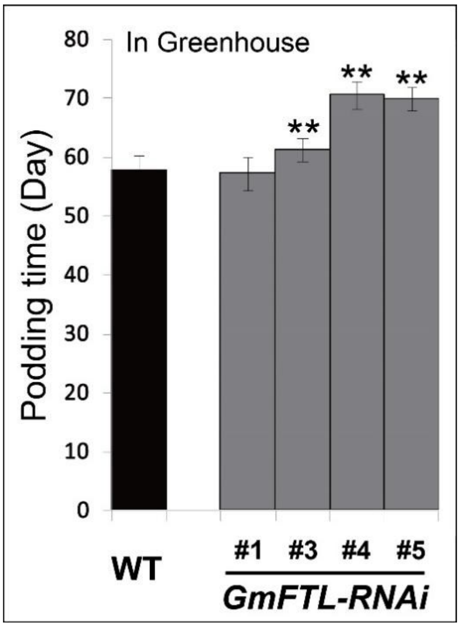

Figure S5 Reducing *GmFTL* expression delays soybean podding time. WT and *GmFTL*-RNAi line #1, #3, #4, and #5 grew in greenhouse. An asterisk indicates significantly difference compared to wild type (**, $P < 0.01$. Student's *t*-test, n ≥ 20 plants).

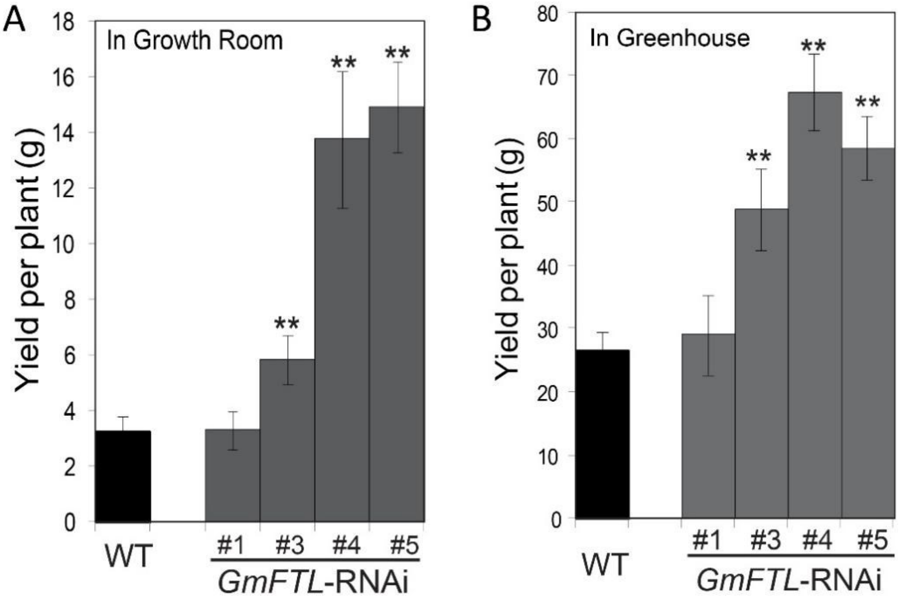

Figure S6 Reducing *GmFTL* expression increases soybean yield. WT and *GmFTL*-RNAi line #1, #3, #4, and #5 grew in growth room and greenhouse. This figure is supporting data for Figure 1. An asterisk indicates significantly difference compared to wild type (**, $P < 0.01$. Student's *t*-test, n ≥ 10 plants).

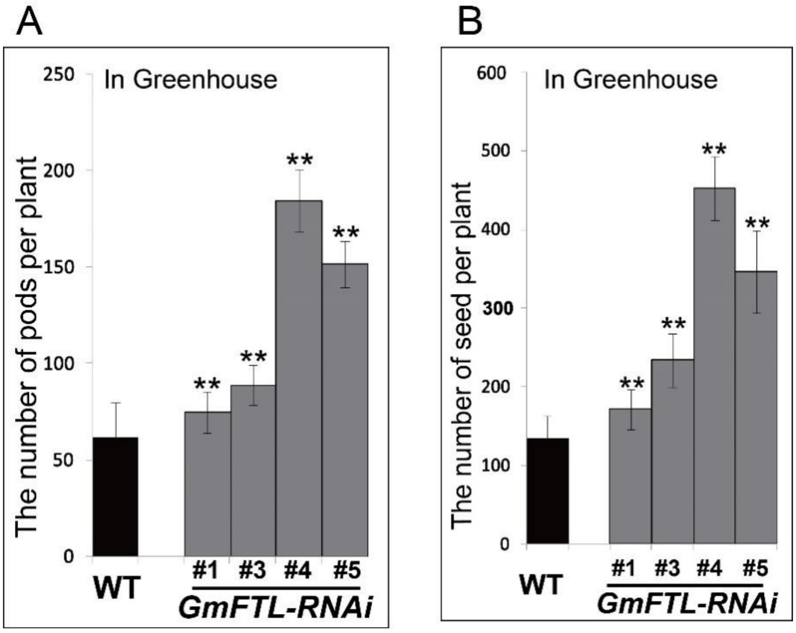

Figure S7 Reducing *GmFTL* expression increases the number of pods and seeds per plant. WT and *GmFTL*-RNAi line #1, #3, #4, and #5 grew in greenhouse. An asterisk indicates significantly difference compared to wild type (**, $P < 0.01$. Student's *t*-test, n ≥ 20 plants).

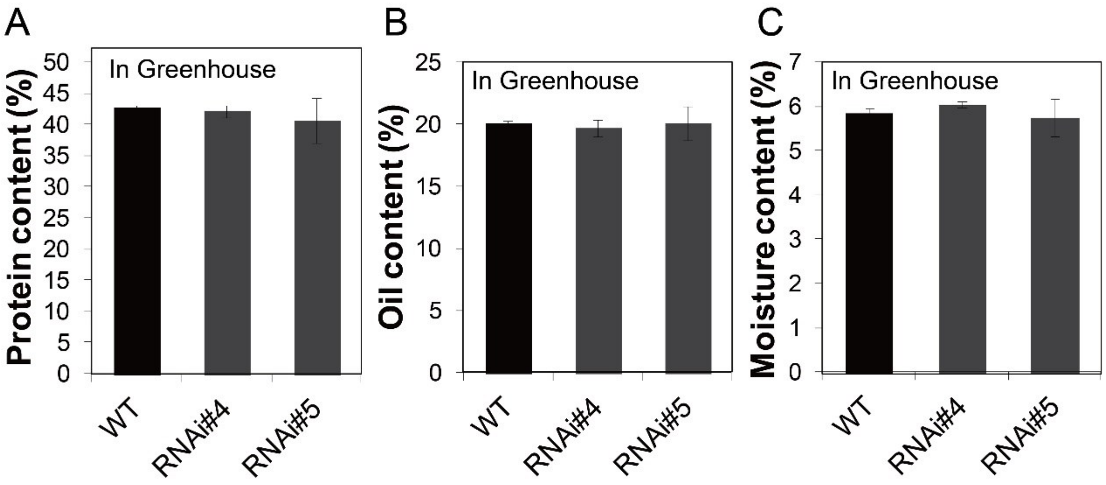

Figure S8 Reducing *GmFTL* expression does not altered the quality of soybean seeds. *GmFTL*-RNAi line #4 and #5 grew in greenhouse have similar contents of proteins (**A**), oils (**B**), and water (**C**) as wild type seeds.

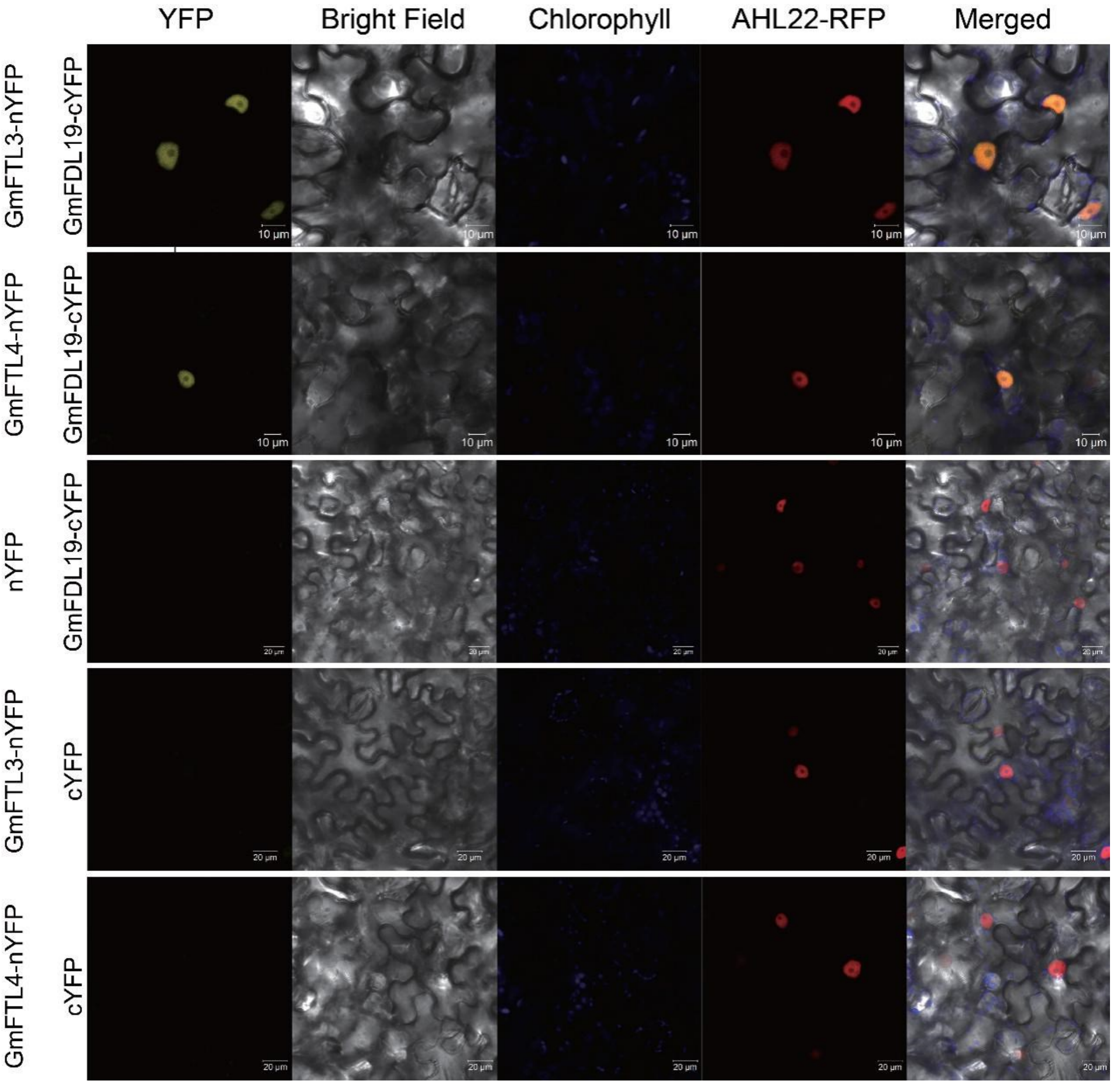

Figure S9 Both GmFTL3 and GmFTL4 proteins interact with GmFDL19 in the nucleus. *Agrobacterium tumefaciens* harboring pEarlyGate201-*GmFTL3:nYFP*, or pEarlyGate201-*GmFTL4:nYFP*, pEarlyGate202-*GmFDL19:cYFP* vectors were co-infiltrated into *Nicotiana benthamiana* leaves, respectively. Then, plants were incubated for 48 hours and subsequently observed by bimolecular fluorescence complementation (BiFC) under a confocal microscope. AHL22-RFP was used as a nucleus marker protein. *GmFTL3:nYFP* and cYFP, *GmFTL4:nYFP* and cYFP, and nYFP and *GmFDL19:cYFP* were used as controls.

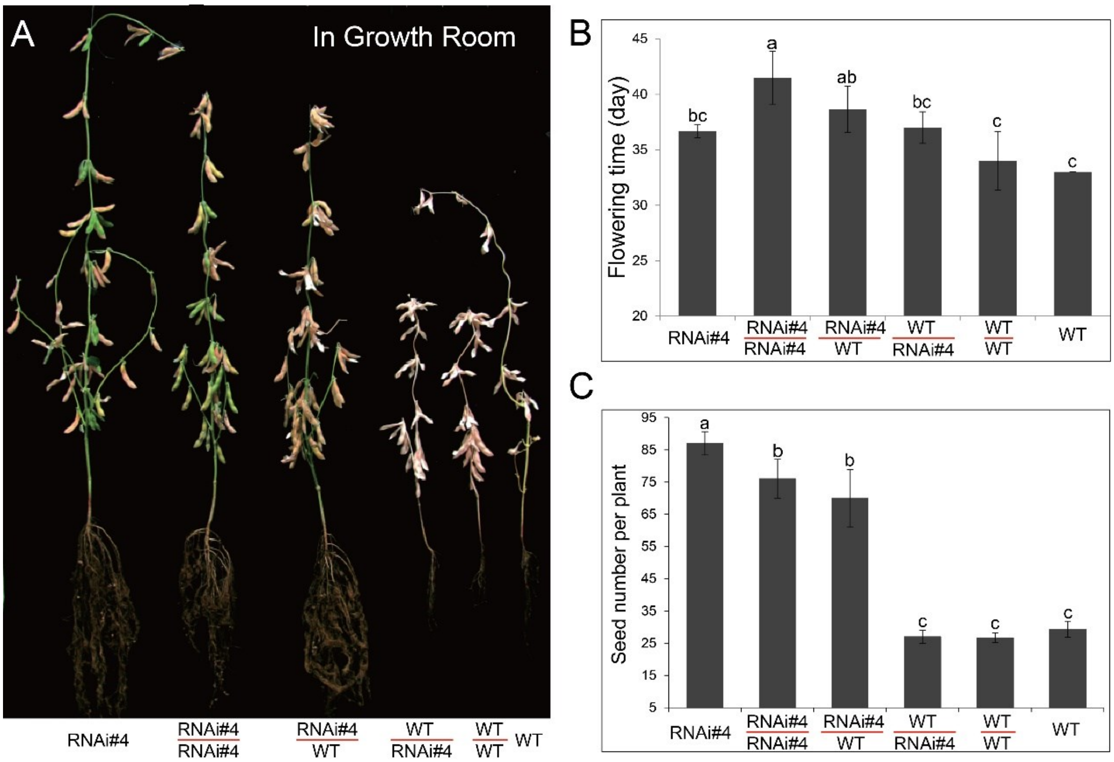

Figure S10 Grafting assay. (**A**), Photos of different graft combinations and ungrafted control plants at maturity. For each graft combination, the genotypes of scion (above) and stock (below) were separated by a red line. (**B**) and(**C**), Flowering time (**B**) and seed number per plant (**C**) for different graft combinations. Statistical significance at the 0.01 level was determined using Duncan's multiple range test, n ≥ 3 plants

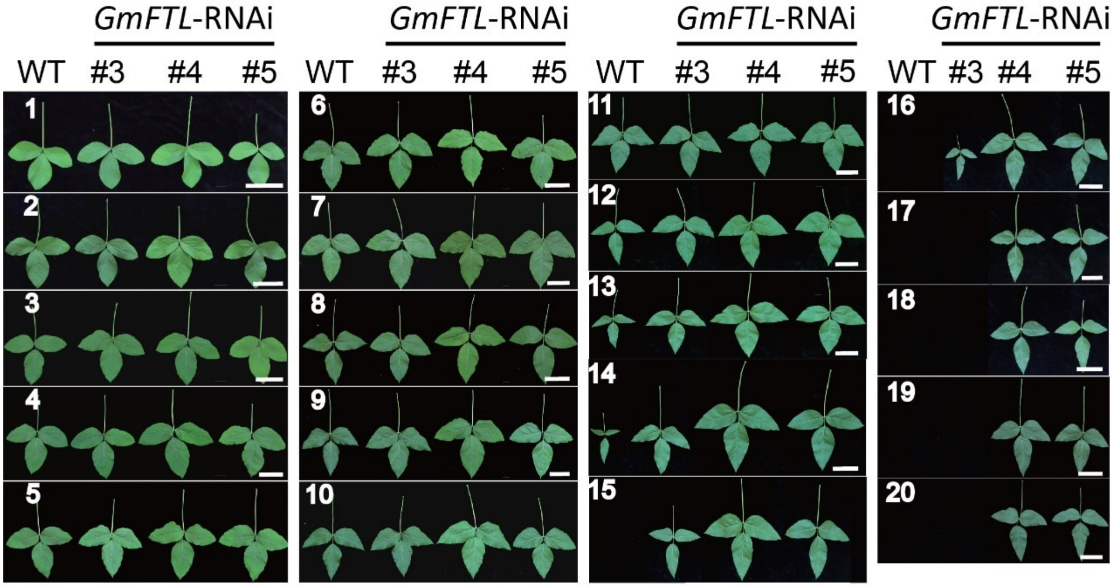

Figure 11 Reduction of *GmFTL* transcripts promotes growth of trifoliolate leaves. This photos shows morphology of trifoliolates of *GmFTL*-RNAi #3, #4, and #5 growing in greenhouse. The leaf order is according to the developmental subsequence of trifoliolates. This figure is a supporting data for Figure 4.

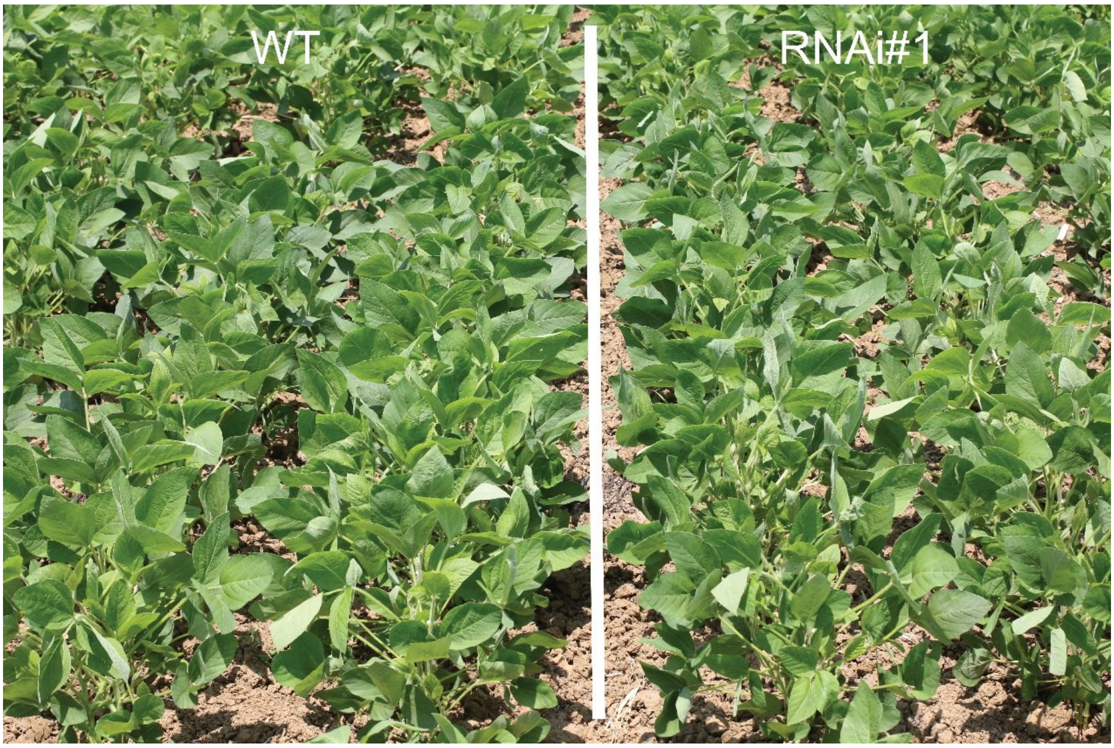

Figure 12 *GmFTL*-RNAi line #1 does not show significant difference at the vegetative stage compared with wild type plants. This photo shows plants of *GmFTL*-RNAi #1 grew in field in Hanchuan, the original location of wild type plants, in 2018. The photo indicates there is no difference between *GmFTL*-RNAi #1 (right) and wild type (left) plants. Seeds were sown at April 20 and photos were token at Jane 5.

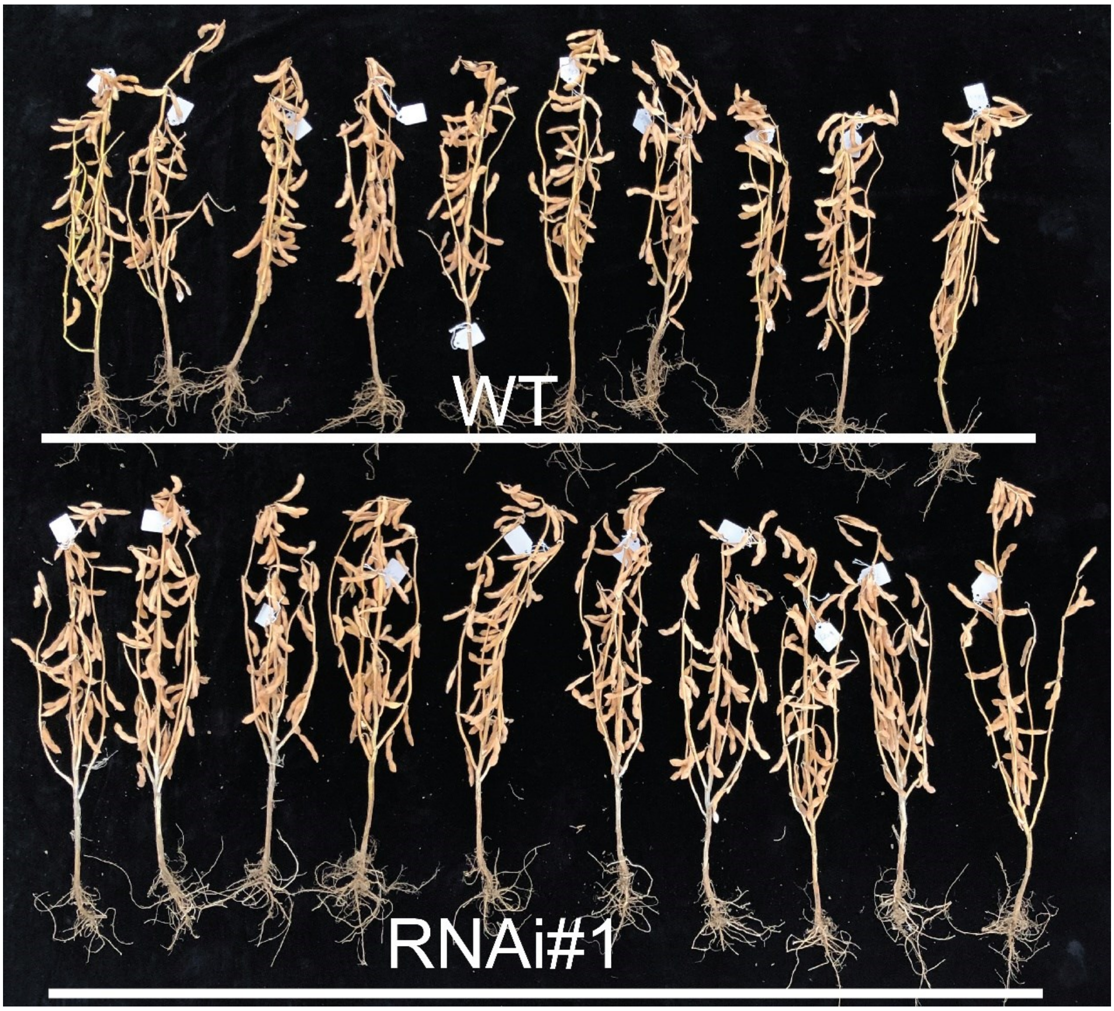

Figure 13 *GmFTL*-RNAi line #1 shows significant difference at the reproductive stage from wild type plants. This photo shows dry plants at maturity of *GmFTL*-RNAi #1 and wild type plants grew in field in Hanchuan, the original location of wild type plants, in 2018. The photo displays that *GmFTL*-RNAi #1 plants (low row) are bigger stature and have more pods than wild type plants (up row).

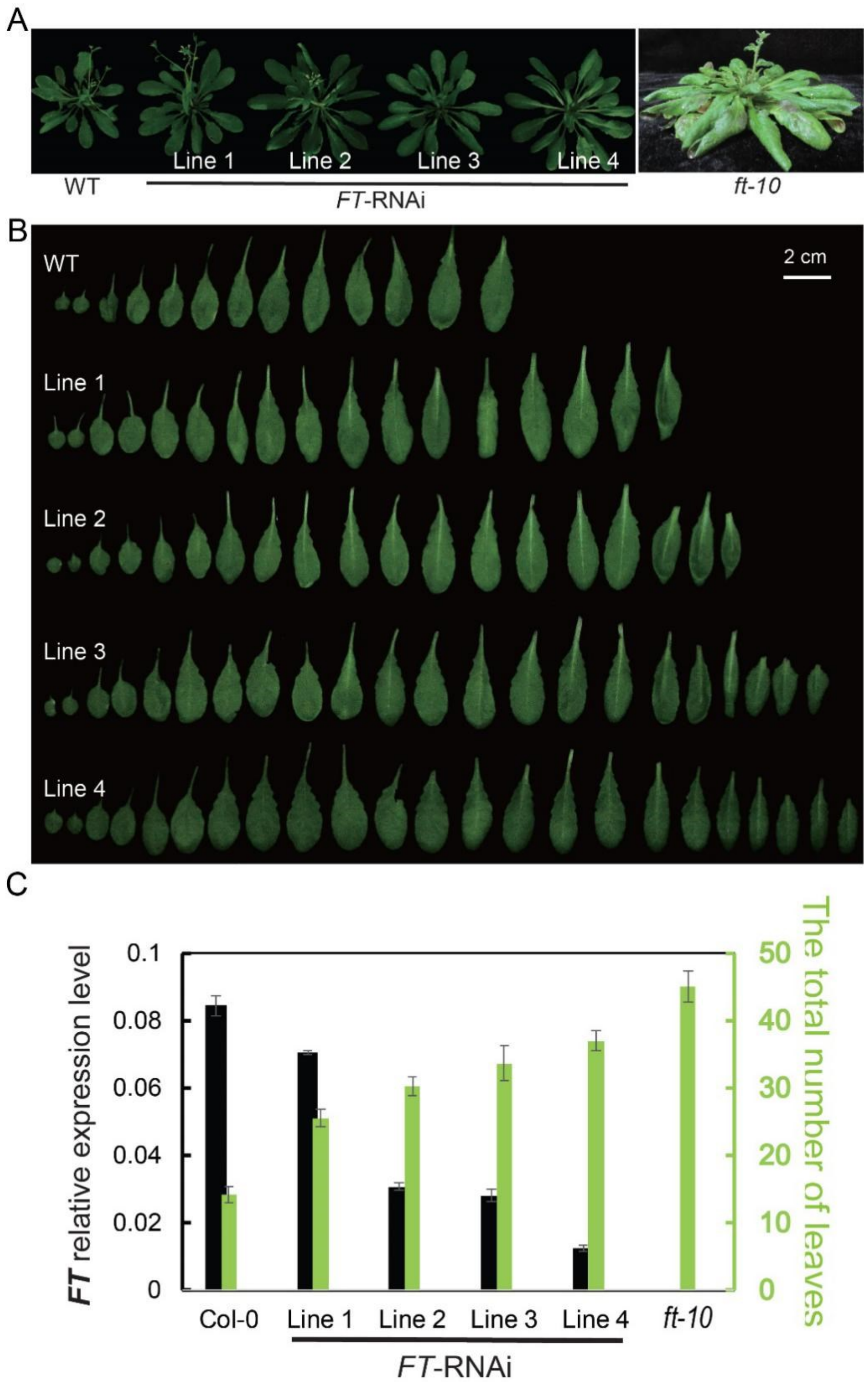

Figure S14 The expression level of florigen is positively related to biomass and leaf size in *Arabidopsis*. *AtFT*-RNAi lines were produced by introducing a RNAi fragment of *Arabidopsis* florigen *FT* gene (At1g65480) into ecotype Columbia-0 and homozygous transgenic lines were used to all experiments. *ft-10* is a null mutant of the florigen *AtFT* gene. *Arabidopsis* plants (wild type, *AtFT*-RNAi lines, and *ft-10* mutants) grew in long day conditions (growth room). Photos of rosettes (**A**) and leaves (**B**) were taken at flowering. The *Arabidopsis* florigen *FT* gene expression was determined by RT-qPCR at day 14 after germination (**C**). The flowering time is negatively correlated to flowering time (**C**) in long day conditions.
