# Supplementary material for: Fine-Tuning Florigen Increases Field Yield Through Improving Photosynthesis in Soybean
Source: Front Plant Sci. 2021 Aug 16;12:710754. doi: 10.3389/fpls.2021.710754 (PMC8415793; doi:10.3389/fpls.2021.710754)
Supplement: Supplementary Figure 1 — GmFTL-RNAi reduces the mRNA abundance of GmFTL3 and GmFTL4 in different transgenic lines. WT and GmFTL-RNAi lines #1, #3, #4, and #5 grew in growth room, and the first trifoliolate leaves were harvested to investigate gene expression at ZT4 by RT-qPCR. GmACT11 was used as a reference gene. Among these transgenic lines, line #1 shows slight change in GmFTL3 and GmFTL4 expressions. Error bars indicate the standard deviation of the mean of three replicates. An asterisk indicates significant difference compared with wild-type plant (∗∗, P < 0.01. Student’s t-test, n ≥ 5 plants). [file Data_Sheet_1.zip › Supplementary Table S1.docx]

**Table S1 Light information in growth room and green house**

| Light spectrum | Light intensity | |
| --- | --- | --- |
|  | **Growth room**  **μmol·m^-2^·s^-1^** | **Greenhouse**  **μmol·m^-2^·s^-1^** |
| 400~700 nm | 298.25 | 201.31 |
| 360~399 nm | 0.46 | 4.53 |
| 400~499 nm | 38.79 | 40.45 |
| 500~599 nm | 65.34 | 86.39 |
| 600~699 nm | 193.64 | 73.97 |
| 700~760 nm | 50.33 | 28.15 |
