# Supplementary material for: Fine-Tuning Florigen Increases Field Yield Through Improving Photosynthesis in Soybean
Source: Front Plant Sci. 2021 Aug 16;12:710754. doi: 10.3389/fpls.2021.710754 (PMC8415793; doi:10.3389/fpls.2021.710754)
Supplement: Supplementary Figure 1 — GmFTL-RNAi reduces the mRNA abundance of GmFTL3 and GmFTL4 in different transgenic lines. WT and GmFTL-RNAi lines #1, #3, #4, and #5 grew in growth room, and the first trifoliolate leaves were harvested to investigate gene expression at ZT4 by RT-qPCR. GmACT11 was used as a reference gene. Among these transgenic lines, line #1 shows slight change in GmFTL3 and GmFTL4 expressions. Error bars indicate the standard deviation of the mean of three replicates. An asterisk indicates significant difference compared with wild-type plant (∗∗, P < 0.01. Student’s t-test, n ≥ 5 plants). [file Data_Sheet_1.zip › Supplementary Table S3.docx]

**Table S3 A list of oligonucleotide and primer sequences used in this study**

| Oligo Name | Sequence (5’-3’) | Purpose |
| --- | --- | --- |
| GmFTL3-F | tgatggggattcatcgtttggtg | RT-qPCR |
| GmFTL3-R | ttagtataacctccttccaccag |  |
| GmFTL4-F | gccttactccagcttatact | RT-qPCR |
| GmFTL4-R | ggcatgctctagcattgcaa |  |
| GmFDL19-F | ctttgtacacacgggttc | RT-qPCR |
| GmFDL19-R | gaagagaagcagcagaga |  |
| Glyma.11G110100-F | gccatataattcttaatcttccct | RT-qPCR |
| Glyma.11G110100-R | gtttaagctacaccagcatcc |  |
| Glyma.14G151400-F | ttcgctgaagctcttgctc | RT-qPCR |
| Glyma.14G151400-R | ggaactacgaagtaccacc |  |
| Glyma.06G067400-F | ggtccacattcttcttgtcttg | RT-qPCR |
| Glyma.06G067400-R | aacaacttggaaggtttcatttc |  |
| Glyma.17G130100-F | gttcgagtaacacgcagag | RT-qPCR |
| Glyma.17G130100-R | tcgttcagtttagcagatttca |  |
| Glyma.09G129000-F | ccagactcggaacagcaa | RT-qPCR |
| Glyma.09G129000-R | cttcaccttcgccatcaac |  |
| Glyma.09G271600-F | tgagccttgtaatgtagtagtt | RT-qPCR |
| Glyma.09G271600-R | cttgtgtcaatgaagcagtg |  |
| Glyma.10G128100-F | tgtagctgctttgcgaaa | RT-qPCR |
| Glyma.10G128100-R | cagagagaggtgcgattg |  |
| Glyma.15G114600-F | acccatttcaaccacatct | RT-qPCR |
| Glyma.15G114600-R | tttctcaataggtaatgttgctc |  |
| Glyma.20G158300-F | tcatataacttgtgaaccgaac | RT-qPCR |
| Glyma.20G158300-R | gcgagacaaaagcgaaag |  |
| Glyma.06G224500-F | tgactatggtacgcaactg | RT-qPCR |
| Glyma.06G224500-R | cacgatgtctaaggacacg |  |
| Glyma.08G281300-F | gtccaaccgttctacaag | RT-qPCR |
| Glyma.08G281300-R | acaccatctgatttcaaagt |  |
| GmACT11-F | tcatttccacttgatccc | RT-qPCR |
| GmACT11-R | caaacaagaggttgaatatcc |  |
| GmFTL3-TF | cattggttggtgactgatat | TaqMan analysis for GmFTL3 |
| GmFTL3-TR | tgcatacacggtctcc |  |
| GmFTL3-probe | cacaaacaccaaacgatgaatccc |  |
| GmFTL4-TF | gctaccacaaatgcaag | TaqMan analysis for GmFTL4 |
| GmFTL4-TR | ttgctggaacaatacgaa |  |
| GmFTL4-probe | tatgagagcccgaacccttcagt |  |
| GmUKN2-TF | agactaaggagcttgaattc | TaqMan analysis for GmUKN2 |
| GmUKN2-TR | gctgacttgacctcttc |  |
| GmUKN2-probe | aactctcaactacttctcgttccatag |  |
| AtFT-RNAi-F | gcaatgagattgtgtgttacg | AtFT-RNAi |
| AtFT-RNAi-R | ctggtgcatacactgtttgc |  |
